# Chinese Herbal Medicine for Primary Liver Cancer Therapy: Perspectives and Challenges

**DOI:** 10.3389/fphar.2022.889799

**Published:** 2022-05-05

**Authors:** Kexin Li, Kunmin Xiao, Shijie Zhu, Yong Wang, Wei Wang

**Affiliations:** ^1^ Graduate School, Beijing University of Chinese Medicine, Beijing, China; ^2^ Dongzhimen Hospital of Beijing University of Chinese Medicine, Beijing, China; ^3^ Department of Oncology, Wangjing Hospital, China Academy of Chinese Medical Sciences, Beijing, China; ^4^ School of Traditional Chinese Medicine, Beijing University of Chinese Medicine, Beijing, China; ^5^ Institute of Prescription and Syndrome, Guangzhou University of Chinese Medicine, Guangzhou, China; ^6^ Guangdong Provinvial Key Laboratory of TCM Pathogenesis and Prescriptions of Heart and Spleen Diseases, Guangzhou, China

**Keywords:** primary liver cancer, Chinese herbal medicines, Chinese patent medicine, clinical trials, pharmacological mechanisms

## Abstract

Primary liver cancer (PLC) is one of the most common solid malignancies. However, PLC drug development has been slow, and first-line treatments are still needed; thus, studies exploring and developing alternative strategies for effective PLC treatment are urgently needed. Chinese herbal medicine (CHM) has long been applied in the clinic due to its advantages of low toxicity and targeting of multiple factors and pathways, and it has great potential for the development of novel natural drugs against PLC.

**Purpose:** This review aims to provide an update on the pharmacological mechanisms of Chinese patent medicines (CPMs) and the latest CHM-derived compounds for the treatment of PLC and relevant clinical evaluations.

**Materials and Methods:** A systematic search of English literature databases, Chinese literature, the Clinical Trials Registry Platform, and the Chinese Clinical Trial Registry for studies of CHMs for PLC treatment was performed.

**Results:** In this review, we summarize the clinical trials and mechanisms of CPMs for PLC treatment that have entered the clinic with the approval of the Chinese medicine regulatory authority. These CPMs included Huaier granules, Ganfule granules, Fufang Banmao capsules, Jinlong capsules, Brucea *javanica* oil emulsions, and compound kushen injections. We also summarize the latest *in vivo*, *in vitro*, and clinical studies of CHM-derived compounds against PLC: icaritin and ginsenoside Rg3. Dilemmas facing the development of CHMs, such as drug toxicity and low oral availability, and future developments are also discussed.

**Conclusion:** This review provides a deeper the understanding of CHMs as PLC treatments and provides ideas for the development of new natural drugs against PLC.

## Introduction

Primary liver cancer (PLC) is the most common primary malignancy. Globally, 910,000 new cases and approximately 0.83 million deaths due to PLC were reported in 2020, with PLC ranking sixth in mortality of highly malignant neoplasms, and more than half of the global liver cancer burden is in China (IARC, 2020; Shi et al., 2021). Hepatocellular carcinoma (HCC) accounts for the majority of pathological cases of PLC. The occurrence of PLC is associated with chronic hepatitis virus infection, long-term exposure to carcinogens, excessive alcohol consumption, nonalcoholic fatty liver, hemochromatosis, and α-1 antitrypsin deficiency (Eriksson et al., 1986; Marengo et al., 2016).

Patients with early-stage PLC can be treated with local surgical resection, liver transplantation, radiofrequency ablation, and transarterial chemoembolization (TACE). However, due to the insidious onset, high degree of malignancy, rapid development, and easy infiltration and metastasis of HCC, most patients with PLC miss the optimal surgical treatment time (El-Serag et al., 2008; Anwanwan et al., 2020). For patients with advanced HCC, the most common systemic drug therapies are tyrosine kinase inhibitors (TKIs) that inhibit angiogenesis, including sorafenib, lenvatinib, and regorafenib, but treatment with TKIs often causes adverse effects, such as diarrhea, rash, fatigue, hand-foot skin reaction, hypertension and decreased appetite. These symptoms seriously affect therapeutic effects (Hartmann et al., 2009; Rimassa et al., 2019). PD-1/PD-L1 immune checkpoint inhibitors have greatly changed tumor therapy. Unfortunately, the overall response rate to immune checkpoint inhibitors in patients with HCC is only 15%–20%, and the immunosuppressive microenvironment of HCC severely hinders the efficacy of existing immunotherapies (El-Khoueiry et al., 2017). Therefore, studies exploring new drugs or adjuvant therapies for the treatment of PLC are important.

Chinese herbal medicine (CHM) is a bountiful untapped resource. For example, artemisinin, a CHM, has been recommended by World Health Organization (WHO) as a first-line treatment for malaria (World Health Organization, 2015). An increasing number of research results show that CHMs may combat liver cancer by inhibiting cell proliferation, inducing cell apoptosis, inhibiting cell migration and invasion, inhibiting angiogenesis, regulating immunity, reversing drug resistance and exerting other effects (Hu et al., 2013). A multicenter, open label, dose escalation phase I/II study in the America revealed that a classical formula of traditional Chinese medicine (TCM), Huangqin Decoction (PHY906), not only showed a favorable safety profile but also exerted beneficial effects when combined with capecitabine in an Asian subgroup of advanced HCC, with Asian patients having a longer median overall survival (mOS) than non-Asian patients (16.5 vs. 6.2 months, respectively; *p* = 0.03) (Yen et al., 2009). The phase II results further showed that PHY906 increases the therapeutic index of capecitabine by enhancing the antitumor activity of capecitabine and reducing its toxicity profile, thereby prolonging the mOS of patients with advanced HCC by 3 months (Changou et al., 2021). Furthermore, a large cohort study with 127,237 liver cancer patients showed that TCM users was significantly associated with a decreased risk of death compared with non-TCM users [hazard ratio (HR) = 0.65, 95% confidence interval (CI) = 0.64–0.66]. The TCM classic formulas Jia Wei Xiao Yao San (HR = 0.89, 95% CI = 0.8–0.96) and Chai Hu Shu Gan Tang (HR = 0.86, 95% CI = 0.78–0.95) improved the survival of patients with HCC (Liao et al., 2015). Chinese herbal compounds are playing an increasing role in improving clinical symptoms, enhancing and attenuating symptoms, enhancing immunity, and improving the survival rate (SR) and quality of life of patients with liver cancer, with the advantages of low toxicity, and targeting multiple factors and pathways (Qi et al., 2015).

In this review, we summarize commonly used Chinese patent medicines (CPMs) and related clinical evaluations, the latest CHM-derived compounds, and important pharmacological mechanisms of CHMs for the treatment of liver cancer, providing insights for studies aiming to explore the pharmacological mechanisms of CHMs and the development of new natural drugs.

## Chinese Patent Medicines in Primary Liver Cancer

**FIGURE 1:**
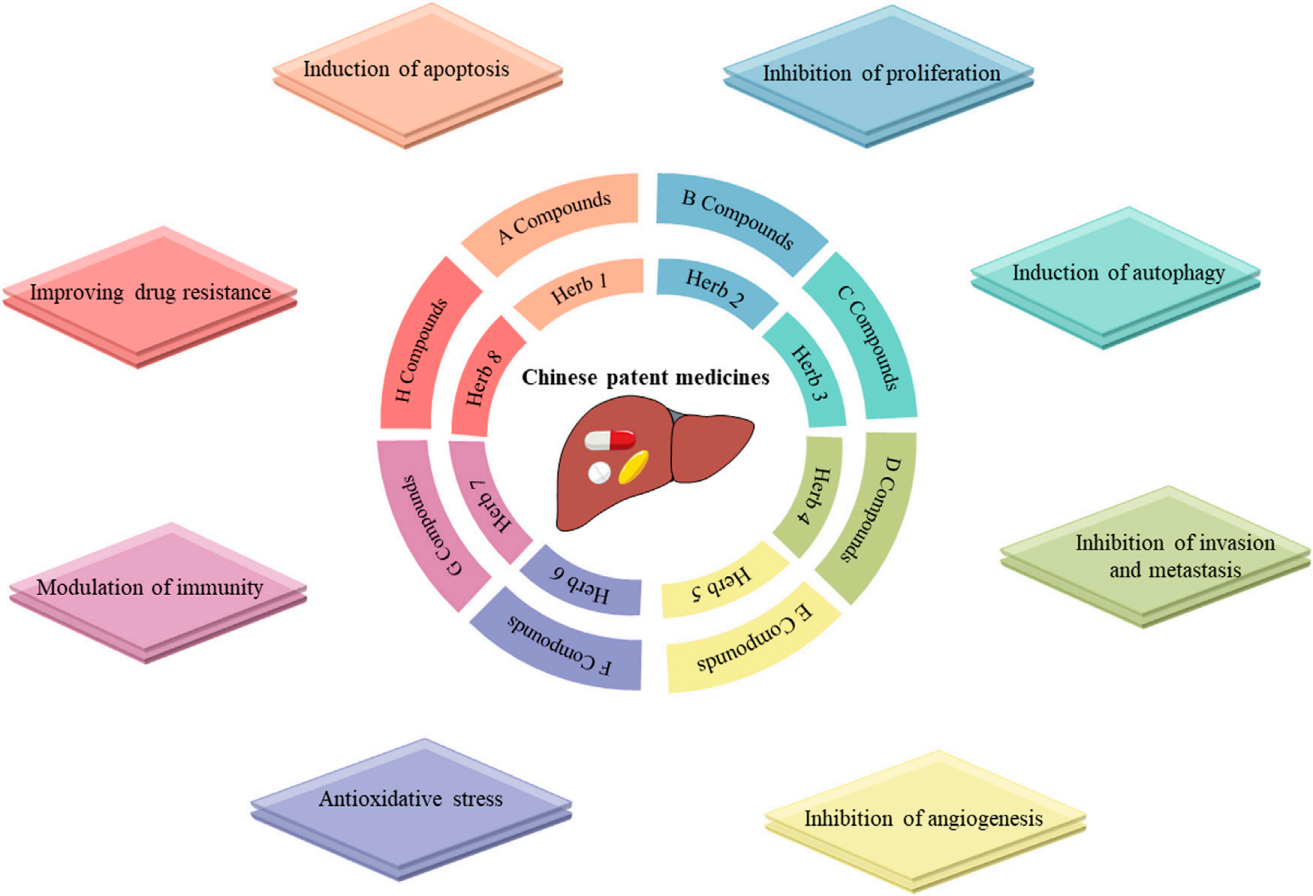
Mechanisms of Chinese patent drugs against primary liver cancer. Notes: Chinese patent medicines often comprise of one or more herbs, each containing various compounds that exert different antitumor activities.

CPMs are traditional Chinese medicinal products processed into a certain dosage form under the guidance of TCM theory and according to prescribed prescription and formulation processes. CPMs tend to be composed of multiple components that are not simply added together at random, but in an orderly manner (Figure 1). CPMs must undergo a scientifically rigorous clinical evaluation and be approved by the Chinese medicine regulatory authority before they enter clinical use. In this section, we describe clinical trials of commonly used CPMs for the treatment of PLC or PLC-related symptoms as well as their mechanisms (Tables 1, 2).

**TABLE 1:** Chinese patent medicines used to treat PLC.

Patent drug name	Dosage forms	Components	Therapeutic efficacy and mechanisms
Huaier granules	Granule	*Trametes robiniophila* Murr	Patient objective remission rate↑, disease control rate↑, survival rate↑, KPS score↑, CD3^+^ T cells↑, CD4^+^ T cells↑, CD8^+^ T cells↑, CD4^+^/CD8^+^ T cells↑, AFP↓, recurrence rate↓, adverse reactions↓ (Zhang et al., 2021); Recurrence free survival↑, extrahepatic recurrence↓ (Chen et al., 2018); Progression-free survival↑, recurrence↓ (Wang Z. et al., 2021); Inhibits proliferation and migration of HCC cells, YAP1↓, PCNA↓, Ki-67↓, CyclinD1↓, Bcl-2↓, Bax↑ (Shan et al., 2017); MCM↓, CDK19↓, CyclinD1↓, CyclinE1↓, p53↑, p21↑, p27↑ (Niu et al., 2020); Caspase 3 ↑ PARP↑, p-ERK↓, p-P38↓, p-JNK↓, cell cycle arrest in S phase↑, β-catenin↓, cyclin D1↓ (Zhang et al., 2015); G0/G1 phase↓, CEACAM1↑, HBx↓ (Zhong et al., 2018); Anti-angiogenesis, HIF-1α/VEGF↓,AUF-1/AEG-1↓, PNCA↓ (Li et al., 2015); Improves chemotherapeutic drug efficacy, YAP↓ (Tao et al., 2018)
Ganfule granules	Granule	Aquilariae Lignum Resinatum (Chenxiang), Cyperi Rhizoma (Xiangfu), Akebiae Caulis (Mutong), Artemisiae Scopariae Herba (Yinchen), Ostreae Concha, Sappan Lignum (Sumu), Curcumae Radix (Yujin), Coicis semen (Yiyiren), *Poria* (Fulin), *Patrinia Scabiosaefolia* (Baijiangcao), Scutellariae Barbatae Herba (Banzhilian), Persicae semen (Taoren), Rhei Radix Et Rhizoma (Dahuang), *Eupolyphaga steleophaga, Citri reticulatae* Pericarpium (Chenpi), Astragali Radix (Huangqi), *Atractylodis macrocephalae* Rhizoma (Baizhu), Paridis Rhizoma (Chonglou), Trionycis Carapax, Codonopsis Radix (Dangshen), Bupleuri Radix (Chaihu)	Patient survival time↑ (Hao et al., 2013); KPS score↑, AFP↓ (Gao, 2014); Improves the gut microbiota structure (Xu et al., 2021)
Fufang Banmao capsule	Capsule	Mylabris (Banmao), Ginseng Radix Et Rhizoma (Renshen), Astragali Radix (Huangqi), Acanthopanacis Senticosi Radix Et Rhizoma Seu Caulis (Ciwujia), Sparganii Rhizoma (Sanleng),Scutellariae Barbatae Herba (Banzhilian), Curcumae Rhizoma (Ezhu), Corni Fructus (Shanzhuyu), Ligustri Lucidi Fructus (Nvzhenzi), Bear bile powder, Glycyrrhizae Radix Et Rhizoma (Gancao)	Patient survival time↑ (Liu et al., 2020); AnxA5↑, HSPA8↑, eIF5A↓, Prdx2↓ (Cao et al., 2007)
Jinlong capsule	Capsule	Fresh gecko, Fresh multibanded krait, Fresh long-nosed pit viper	Lymphocyte function↑, IL-2 ↑, slL-2R↓ (Zhang et al., 2008); Overall survival↑, overall response rate ↑, disease control rate↑, adverse events related to TACE↓ (Jia et al., 2020); Short-term clinical efficacy, life quality↑, OPN↓, KPS score↑ (Wu et al., 2010)
Brucea *javanica* oil emulsion	Emulsion	Refined Brucea *Javanica o*il, Refined soybean phospholipid, Glycerol	Patient response rate↑, survival rate↑, sFas/sFasL↓ (Jin et al., 2015); Inhibits proliferation of HepG2, decreases the intrahepatic metastasis rate (Yue et al., 2015); Induces apoptosis in Hep3B spheroids, promotes apoptosis, PARP↓, Akt↓, caspase-3↓, cell cycle arrest in G1↑ (Chen et al., 2016); Akt↓, TGF-β1↓,α-SMA↓ (Shi et al., 2015); Induces H22 cells apoptosis, miRNA-29b↑, p53↑, Bax ↑, Bad↑, Bcl-2↓, mitochondrial Cytochrome C↓, cytosol Cytochrome C↑, cleaved-caspase 3↑, cleaved-caspase 9↑, PARP↑, cleaved- PARP↑ (Wang T. et al., 2021)
Compound kushen injection	Injection	Sophorae *flavescentis* Radix (Kushen), Smilacis Glabrae Rhizoma (Tufuling)	Reshapes the immune microenvironment, TNFR1↑, CD8^+^ T↑, improves chemotherapeutic drug efficacy (Yang et al., 2020); Anti-angiogenesis (Wang H. et al., 2019); Metabolic reprogramming, Wnt/β-catenin pathway↑, c-Myc↓ (Wang K. X. et al., 2021a); EGFR-STAT3 signaling pathway c-Myc↓ (Wang K.X. et al., 2021b); Cell cycle↓, energy metabolism↓, DNA repair pathways↓ (Cui et al., 2019); Inhibites the proliferation of HepG2 cells, SRD5A↑, ADH1A↑, CDK1↓, EPHX2↓ (Lu et al., 2022)

**TABLE 2:** Chemical analysis of Chinese patent medicines.

Patent drug name	Analytical Methods	Main chemical composition	References
Huaier granules	UHPLC-MS	Proteoglycan,1β-Hydroxyalantolactone, 2-Aminoisobutyric acid, 2-Hydroxybutanoic acid, 2-Isopropyl-3-oxosuccinate, 2′-O-Methyladenosine, 2-Picolinic acid, 3,4-Dihydroxyphenylacetaldehyde, 3-Ethyl-1,2-benzenediol	Pan et al. (2019), Jian et al. (2022)
Ganfule granules	UPLC-Q-TOF-MS	Chlorogenic acid, amygdalin, 3′-deoxysappanone A, 10-O-Methylprotosappanin B, scutellarin, narirutin, hesperidin, hesperetin, nobiletin, 3,3′,4′,5,6,7,8-heptamethoxyflavone, saikosaponin A, Saikogenin C and astragaloside I	Xu et al. (2021)
Fufang Banmao capsule	GC	Cantharidin, ginsenosides Rg1, ginsenosides Rb1, ginsenosides Re, astragaloside A, isofraxidin	Mi et al. (2003)
Jinlong capsule	UPLC	Histidine, serine, arginine, glycine, aspartic acid, glutamate, threonine, alanine, proline, cystine, lysine, tyrosine, methionine, valine, isoleucine, leucine, phenylalanine	LI and Xiao, (2014)
Brucea *javanica* oil emulsion	HPLC, GC-MS	Brusatol, bruceine D, bruceine H, yadanzioside A, yadanzioside G, javanicoside C and bruceantinoside A, phenol, hexadecanoic acid, octadecanoic acid, 9-Octadecenoic acid (oleic acid), 9E,12Z-Octadecadienoic acid (linoleic acid), 9Z,12Z,15Z-Octadecatrienoic acid	Chen et al. (2009), Zhang et al. (2011), Zhao et al. (2011), Tongtong Wang et al. (2021)
Compound kushen injection	UPLC-MS, HPLC	Adenine, N-methylcytisine, sophorodine, matrine, sophocarpine, oxysophocarpine, oxymatrine, trifolirhizin	Qi et al. (2013), Gao et al. (2018), Shen et al. (2019)

UPLC-MS, ultra-performance liquid chromatography/mass spectrometry; UPLC-Q-TOF-MS, ultra-performance liquid chromatography to quadrupole time-of-flight mass spectrometry; GC, gas chromatography; HPLC, liquid chromatography–mass spectrometry; GC-MS, gas chromatography-mass spectrometry; UHPLC-MS, ultrahigh-pressure liquid chromatography coupled with tandem mass spectrometry.

### Huaier Granules

Huaier granules are one of the most widely used CPMs for the treatment of liver cancer (Lei et al., 2015; Chen et al., 2018). *Trametes robiniophila* Murr, the main component of Huaier granules, is a medicinal fungus that contains a variety of organic components and more than 10 minerals, and its active component is polysaccharide protein (Pan et al., 2019). *In vitro* and *in vivo* studies have shown that Huaier granules target multiple factors and pathways in the treatment of liver cancer (Pan et al., 2019; Qi et al., 2020). Huaier granules inhibit the proliferation of HCC cells by inhibiting six minichromosomes and Yes-associated protein 1 (Ren et al., 2009; Zhang et al., 2015; Shan et al., 2017; Niu et al., 2020), and induce HepG2 cell apoptosis by upregulating HBx gene expression and downregulating CEACAM1 gene expression (Zhong et al., 2018). Downregulation of HIF-1alpha/VEGF and AUF-1/AEG-1 signaling interferes with tumor angiogenesis (Li et al., 2015; Zou et al., 2015). Furthermore, Huaier granules improve chemosensitivity and reverse the resistance of liver cancer to oxaliplatin by downregulating YAP (Tao et al., 2018).

A randomized, controlled, phase IV trial was conducted at 39 hospitals in China from 2011 to 2014, and 1044 postoperative patients who underwent curative resection of HCC were enrolled and divided into a treatment group (oral Huaier granules, 20 g three times a day) and a blank control group at a 2:1 ratio. After 96 weeks of follow-up, the study found that Huaier granules had excellent performance in postoperative treatment: the recurrence rates of the Huaier granules group and control group were 37.6% and 50.9%, respectively, representing a 13% difference. In terms of relapse-free survival (RFS), patients in the Huaier granules group had an average RFS of 75.5 weeks, which was 33% longer than that of the control group. In addition, the RFS and overall survival (OS) rates of the Huaier granule group that received treatment for 96 weeks were 62.39% and 95.19% respectively. Regardless of whether patients started to take medicine with or without hepatitis B virus (HBV) infection, cirrhosis, ascites, and/or other conditions, the effect of Huaier granules was quite stable. Regrettably, a placebo was not used in this trial because the bitter taste of Huaier granules is obvious and easily distinguishable, making it difficult to produce a placebo (Chen et al., 2018).

A single center randomized controlled study was conducted to evaluate the safety and efficacy of TACE combined with Huaier granules for the treatment of PLC. Sixty-two patients with PLC were included, and the experimental group was treated with oral Huaier granules, TACE and lobaplatin. The 6-months (87.1% vs. 73.3%) and 1-year objective response rates (72.4% vs. 64.3%) in the experimental and control groups were significantly different; the 6-months and 1-year OS rates were 100% vs. 90.3% and 93.5% vs. 80.6% in the two groups. However, the difference in the 6-months OS rate was not significant. The median survival of the experimental group was 20.6 months, which was 3.5 months longer than that of the control group, and the treatment significantly reduced the number of TACE procedures needed. In addition, a statistically significant difference in the occurrence of adverse events (AEs) was not observed between the two groups. TACE combined with Huaier granules had a certain clinical efficacy for the treatment of PHC; however, large multicenter randomized controlled trials are still needed to confirm the safety and efficacy of this treatment option (Zhao et al., 2017).

Thermal ablation is also an important treatment for patients with early-stage HCC, and 340 patients with early-stage HCC were included in one retrospective study evaluating the prognostic value of Huaier granules for patients with HCC undergoing thermal ablation. The control group was treated with thermal ablation, and the experimental group was treated with thermal ablation combined with Huaier granules. This study confirmed that administration of Huaier granules after ablation improved the 1-, 3-, and 5-years PFS rates (78.8% vs. 69.4%, 50.6% vs. 40.6%, and 35.3% vs. 26.5%, respectively; *p* = 0.020) as well as the rate of extrahepatic metastases [*p* = 0.018, HR = 0.49 (0.27–0.89)], and the benefit obtained was greater with continuous oral intake for more than 2 years. In addition, a significant difference in the incidence of side effects was not observed between the two groups. However, no significant improvement in patient OS was identified. More importantly, patients with any two of the following three factors were predicted to potentially benefit from treatment with Huaier granules: age less than 65 years old, a single tumor, and tumor size ≤3 cm (Wang Z. et al., 2021). In conclusion, the use of Huaier granules as an adjuvant treatment for PLC have certain efficacy and a high safety profile, but the conclusions still require further validation in multicenter, large-sample, randomized, double-blind, controlled, high-quality studies (Zhang et al., 2021).

### Ganfule Capsule

Ganfule capsule (GFL) is a compound preparation composed of 21 herbs (Table 1). Thirteen key compounds were identified in GFL: chlorogenic acid, amygdalin, 3′-deoxysappanone A, 10-O-methylprotosappanin B, scutellarin, narirutin, hesperidin, hesperetin, nobiletin, 3,3′,4′,5,6,7,8-heptamethoxyflavone, saikosaponin A, saikogenin C, and astragaloside I. The anti-HCC effects of GFL are closely related to lipid metabolism, carbohydrate metabolism, and metabolism of multiple amino acids and may affect the structure and composition of intestinal flora, inducing the enrichment of a large number of probiotics strains, and *Lactobacillus*, reducing oxidative stress, promoting apoptosis of liver cancer cells, and inhibiting tumor growth (Xu et al., 2021).

GFL, a complementary alternative therapy, plays an important role in the comprehensive treatment of PLC. One study explored the therapeutic efficacy of GFL combined with chemotherapy in patients with PLC, and 58 patients with advanced HCC were divided into a GFL combined with chemotherapy group and a chemotherapy alone group for a prospective randomized controlled study. The rate of improvement in the Karnofsky performance status (KPS) score in the GFL combined with chemotherapy group was 43.33%. This result was significantly higher than that of the control group (21.43%), indicating that oral GFL improved the quality of life of patients. In addition, the decrease in the serum AFP level in the GFL combined with chemotherapy group was also more significant than that in the chemotherapy alone group, but the treatment response rate (RR), disease control rate (DCR) and the 1-year SR were not significantly different (Gao, 2014). Another study explored the efficacy of TACE combined with GFL for advanced HCC. Sixty-five patients with advanced HCC were treated with TACE combined with GFL, and 67 patients with HCC were treated with TCM alone. The mOS of the TACE combined with GFL group was 205 days, which was significantly higher than the 127 days of the TCM alone group. The 6-months, 1-year, and 2-years OS rates were 58.9, 29.1, and 7.7%, respectively, for the TACE combined with GFL group compared with 33.3%, 12.3%, and 1.8%, respectively, for the TCM alone group, but evidence supporting this combination regimen is still low, and further exploration is needed (Hao et al., 2013).

### Fufang Banmao Capsule

Fufang Banmao capsule (FFBM) was selected as one of the best cancer drugs by the China Food and Drug Administration in 2002; it includes 11 traditional Chinese medicines, of which the main drug is Banmao, and the active ingredient of Banmao is cantharidin (Wang, 1989; Naz et al., 2020). Cantharidin has great anticancer potential and is an inhibitor of protein phosphatase 2A (PP2A), blocking G2/M transformation through the JNK/Sp1 signaling pathway and upregulation of Myt1, p53, histone H2AX, cyclin A2 and cyclin B1 (Gong et al., 2015; Le et al., 2016). Cantharidin has been shown to block the transcriptional activity of heat shock factor 1 (HSF1) and inhibit the expression of heat shock protein 70 and Bcl-2 related athanogene domain 3, thus inducing cancer cells death (Kim J A. et al., 2013).

Clinical studies have shown that compared with the simple supportive treatment group, the addition of FFBM prolongs the 6-months OS (2.2 vs. 4 months) of end-stage patients with Vp3-4 portal vein tumor thrombosis (PVTT) and HCC, especially high-risk patients (score ≥84). These patients often have serious liver function damage and/or serious complications, are unable to receive conventional treatment and require effective adjuvant therapy. FFBM is preferred as an adjuvant drug against cancer because it has no significant side effects and is an independent predictor of OS. However, the study was retrospective; therefore, a rigorous prospective randomized controlled trial is needed to verify the effect of FFBM on the survival of patients with HCC (Liu et al., 2020).

### Jinlong Capsule

Jinlong capsule (JLC), an animal-derived CPM prepared using modern biochemical separation technology involving low temperature and freezing, consists of 17 amino acids and a variety of bioactive peptides (Li et al., 2022), which have significant anticancer activity and improve the immune function and quality of life of patients with various cancers (Jia et al., 2020; Xu et al., 2020).

Two meta-analyses evaluated the efficacy and safety of JLC as an adjuvant drug for the treatment of HCC. The intervention modalities included in these two meta-analyses were distinguished: one study compared the clinical outcomes of adjuvant TACE plus JLC versus TACE alone, and the other evaluated the clinical outcomes of JLC combined with conventional therapy versus conventional therapy alone. Conventional treatments include not only TACE but also chemotherapy, radiotherapy, radiofrequency ablation, percutaneous ethanol injection, supportive and symptomatic treatment, and ultrasound. The results of both of these studies showed that compared with the monotherapy treatment group, the combined treatment group had increased percentages of CD3 ^+^, CD4+and natural killer (NK) cells, an increased ratio of CD4+/CD8+cells, enhanced immune function, and significantly improved overall response, DCR, quality of life (QoL), and OS at 6, 12, 24 and 36 months (Xu et al., 2020). In terms of safety, both meta-analyses showed that JLC effectively reduces AEs, such as leukopenia, gastrointestinal adverse reactions, hepatotoxicity and bone marrow suppression (Jia et al., 2020; Xu et al., 2020). Compared with TACE treatment alone, JLC combined with TACE treatment did not significantly reduce AFP, but it exerted a good regulatory effect on prognostic biomarkers of new liver cancer, such as IL-2, slL-2R and OPN. Notably, the populations included in these two meta-analyses were Chinese, and thus ethnic and regional biases should be considered (Zhang et al., 2008; Wu et al., 2010).

### *Brucea Javanica* Oil Emulsion

The main ingredient of the *Brucea javanica* oil emulsion (BJOE) is *Brucea javanica* oil (BJO), which is prepared using glycerol as the raw material and natural soybean lecithin as the emulsifier. The main active components of BJOE are linoleic acid and oleic acid, which can inhibit the growth of tumor cells (Zhu et al., 1989; Chen et al., 2009; Zhang et al., 2011). *In vitro* studies revealed that BJO not only significantly inhibited the proliferation of HepG2 and Hep3B cells but also participated in p53-, Bax-, Bad-, and Bcl-2-mediated apoptosis and downregulated the expression of the stem cell markers CD133, Nanog, and EpCAM (Yue et al., 2015; Chen et al., 2016). *In vivo* studies revealed that BJO significantly reduced the tumor size and prolonged survival in H22 tumor bearing mice (Shi et al., 2015; Wang T. et al., 2021).

The short-term efficacy of BJOE as an adjuvant drug combined with TACE in the treatment of PLC was not only significantly higher than that of TACE alone (78.12 vs. 50%), but the 1-, 2-, and 3-years SRs of patients treated with the combination were also better than those of patients treated with TACE alone (79.7% vs. 59.4%, 52.3% vs.20.3%, and 23.0% vs. 6.8%, respectively). Furthermore, BJOE combination therapy can reduce the incidence of adverse reactions such as fever, nausea and vomiting. The combination therapy may cooperatively regulate the expression and release of sFas/sFasL in patients with liver cancer, promote liver cancer cell apoptosis and inhibit tumor growth (Jin et al., 2015; Zhang D. et al., 2020). However, no unified specification has been established for the time, dose, dosage form and method of administration of BJO in clinical applications. For example, unified guidelines for whether BJO should be injected before or after interventional chemoembolization or at the same time are unavailable, and no relevant literature has been reported.

BJOE is widely used in clinic. However, BJOE has a multidirectional dynamic unstable dispersion system; an improper temperature can cause separation. its preparation is complex, and its effective components are poorly soluble in fat, leading to low bioavailability in clinical application (Yang et al., 2014; Yoon et al., 2020). Therefore, studies exploring more effective, stable and efficient dosage forms are needed. New dosage forms of BJO include gastroretentive floating bead, self-microemulsion, nanoemulsion, liposomes, nanostructured lipid carriers, and sponges (Yoon et al., 2020).

BJO gastroretentive floating bead consists of alginate and carrageenan (Zhang et al., 2018a). Gastroretentive floating bead allow drugs to remain floating in the stomach for a long time, prolonging the effect of drugs in the stomach and thus improving drug bioavailability (Lopes et al., 2016). Compared with BJO commercial products, the absorption and oral bioavailability of BJO gastroretentive floating bead are enhanced, and it shows better ability in inhibiting cancer cells and preventing gastric ulcer. However, it is still difficult to achieve stable and reliable requirements because gastroretentive floating bead are greatly affected by gastric emptying and food (Zhang et al., 2018b).

The self-microemulsifying drug delivery system are emulsions with particle size less than 100 nm composed of an oil, non-ionic surfactants, cosolvent and drugs. The self-microemulsifying drug delivery system is suitable for use as a carrier for lipophilic, low water solubility, poorly absorbed, easily hydrolyzed drugs (Akula et al., 2014). BJO self-microemulsifying drug delivery system is a fine oil-in-water microemulsion with particle size <50 nm, which not only enhanced the release of BJO but also significantly inhibited the viability of cancer cells and tumor growth in tumor-bearing mice. However, caution has to be exercised as the introduction of a large amount of surfactant reduces the absorption of BJO and limits its use (Shao et al., 2013).

Liposomes are mainly composed of phospholipids and cholesterol with fewer surfactants, which are biocompatible and biodegradable. Compared with BOJE, the average residence time and elimination half-life of BJO loaded liposomes in blood and tissues were increased by 2.8 - and 4.0-fold respectively, while the clearance was decreased by 0.5-fold. Moreover, BJO loaded liposomes were less toxic than BOJE, and the IC (Shi et al., 2015) value of BJO loaded liposomes was only one third of that of BOJE (Cui et al., 2010). *In vitro* study showed that BJO loaded liposomes inhibited the proliferation of HepG2, SMMC-7721 and A2780/DDP cells. BJO loaded liposomes was also shown similar results and prolonged survival time in *in vivo* models of ovarian carcinoma tumor-bearing nude mice, as well as Lewis lung carcinoma, and HepG2 liver carcinoma tumor-bearing mice. However, liposomes may undergo particle aggregation or hydrolysis during long-term storage, and its low level of drug loading may limit the application of BJO (Cui et al., 2010; Yue et al., 2015; Ye et al., 2016).

Nanomedicine delivery strategies play an important role in BJO delivery and mainly include cationic nanoemulsions, nanostructured lipid carriers, sponge-type nanocarriers. BJO cationic nanoemulsions was prepared from BJO, in oil phase, with chitosan as cationic inducer. The oral bioavailability of BJO cationic nanoemulsions was increased by 1.6-fold compared to BJOE. In human lung adenocarcinoma cell line A549 nude mice orthotopic xenograft model, BJO cationic nanoemulsions enhanced the ability of BJO to reduce tumor size itself and in combination with first-line antitumor drugs. It contributed to the possibility of reduced of dosage of BJO for similar effects (Liu et al., 2016). BJO-loaded nanostructure lipid carriers mainly consist of solid and liquid phase lipids, and has high entrapment efficiency and drug loading capacity, up to 99.2% and 10.4%, respectively. BJO-loaded nanostructure lipid carriers still exhibited high stability after 30 days of storage. Additionally, BJO-loaded nanostructure lipid carriers significantly induce apoptosis in human lung cancer cells (Lv et al., 2016). Zou et al. successfully configured BJO sponge-type nanocarriers using the amphiphilic lipid glycerol monooleate and the amphiphile polysorbate 80. When 2 mg/ml BJO solubilized in the lipid dispersion, bjo sponges BJO sponge-type nanocarriers showed the best encapsulation efficiency, drug loading and stability. The IC50 values of BJO sponge-type nanocarriers were 1.8–2.4 times lower than BJO, but significantly increased the number of apoptotic cancer cells, which indicated that BJO sponge-type nanocarriers improve the bioavailability of BJO through sustained release (Zou et al., 2017). The nanomedicine delivery strategies improve the efficiency of BJO. However, due to the high cost of nano materials, large-scale production into clinical application is still impossible. Therefore, new dosage forms still need to be developed and improved. Application of new technologies, new materials, and a combination of multiple formulation techniques are the future directions for the development of new dosage forms of BJO (Table 3).

**TABLE 3:** Status of new dosage forms of BJO.

Dosage forms	Development status	Main Effects	References
Gastroretentive floating bead	*In vivo* treatment efficacy in rat models; *In vitro* drug release and cancer cell inhibition	1. BJO gastroretentive floating bead had a greater treatment performance against gastric cancer cells *in vitro*	Zhang et al. (2018a), Zhang et al. (2018b)
		2. BJO gastroretentive floating bead had the effects of preventing and treating gastric ulcer *in vivo* compared to the BJO marketed production, along with a greater bioavailability and longer drug residence time in the stomach	
Self-microemulsion	*In vitro* cytotoxicity effects and drug release; *In vivo* antitumor effects in S180 sarcoma-bearing mice	1. BJO self-microemulsifying drug delivery system significantly inhibited the viability of cancer cells *in vitro* and the tumor growth of S180 tumor bearing mice	Shao et al. (2013)
		2. Self-microemulsifying drug delivery system enhanced the release of BJO	
Cationic nanoemulsions	*In vivo* treatment efficacy in orthotopic mouse model of lung cancer	1.BJO cationic nanoemulsions increased oral bioavailability and enhanced antitumor effect compared to BOJE	Liu et al. (2016)
		2. BJO cationic nanoemulsions has synergistic effects with first-line antineoplastic drugs	
Liposomes	*In vivo* cytotoxicity effects and pharmacokinetic in mouse and rat models; *In vitro* cancer cell inhibition	1. BJO loaded liposomes were less toxic than BJOE.	Cui et al. (2010), Yue et al. (2015), Ye et al. (2016)
		2. BJO loaded liposomes prolong the drug circulation in the blood and tissues for a longer period of time compared to the BJOE	
		3. BJO loaded liposomes showed higher therapeutic effects than BOJE on cancer cells *in vitro* and mouse tumors *in vivo*	
Nanostructured lipid carriers	*In vitro* cytotoxicity effects and drug release	1. BJO loaded nanostructure lipid carriers prolonged BJO release and enhance the cytotoxicity of BJO	Lv et al. (2016)
		3. BJO loaded nanostructure lipid carriers showed high physical stability after 30 days storage	
		4. BJO loaded nanostructure lipid carriers exhibited high entrapment efficiency and drug loading	
		5. BJO loaded nanostructure lipid carriers improved antitumor efficacy	
Sponges	*In vitro* cytotoxicity effects and drug release	1. BJO sponge-type nanocarriers increased bioavailability and cancer cell inhibition compared to BJO	Zou et al. (2017)

### Compound Kushen Injection

The compound kushen injection (CKI) is extracted and refined as a 7:3 ratio of Radix *Sophorae* flavescentis (kushen) to *Smilacis glabrae* Rhizoma (tufuling) (Wang et al., 2015). Matrine, sophoridine, oxymatrine and oxysophocarpine are the main components of CKI (Qi et al., 2013). CKI dose-dependently inhibits tumor volume, decreases the microvessel density (MVD), and improves the vascular maturity index (VMI) in HepG2 tumor bearing nude mice, and the antiangiogenic effects of CKI may be important in the treatment of HCC (Wang H. et al., 2019). Metabolomics analysis revealed that CKI exerts anti-HCC effects by regulating key pathways of glucose and amino acid metabolism. Twenty-two differentially abundant metabolites were identified after CKI treatment of SMMC-7721 cells, including 16 differentially abundant metabolites in cells and 10 differentially abundant metabolites in culture medium. CASP3, MYC, MMP2, QDPR, GABRE and REG1A were predicted to be the key targets of CKI (Gao et al., 2018). In addition, CKI suppressed c-Myc expression by regulating the Wnt/β-catenin pathway in DEN-induced HCC rats, thereby inhibiting the production of key metabolites (citrate and lactate) and activity of enzymes (HK and PK) in the glycolytic process to regulate metabolic reprogramming in HCC (Wang K. X. et al., 2021b). Ke-Xin Wang’s team and others used the propagation model of developed by Dijkstra to decode the effective network of key genotype-phenotypes for CKI treatment of HCC and found that CKI affect arginine and proline metabolism, aminoacyl-tRNA biosynthesis, D-glutamine and D-glutamate metabolism, alanine, aspartate and glutamate metabolism, and thiamine metabolism. Moreover, EGFR was identified as a core target of the anti-HCC action of CKI, and targeting of EGFR restored metabolic function *in vivo* (Wang K. X. et al., 2021a).

A meta-analysis of 1,338 patients with unresectable HCC showed that compared with TACE alone, CKI combined with TACE significantly improved the tumor response (TR) (OR = 1.84), KPS score (OR = 2.37), Child-Pugh score (OR = 1.81), and 1-year and 2-years SR in patients with HCC (OR = 2.40 and; OR = 2.49, respective). Although a significant difference in 3-years SR was not observed, the combination of CKI with TACE resulted in fewer AEs (Ma et al., 2016). However, this meta-analysis has limitations. Firstly, the number of included studies was small and only 18 studies were included. Secondly, the populations included in the study were all Chinese and could not be representative of the whole population. Thirdly, publication bias may exist because the included literatures were all published in Chinese. Finally, not all studies strictly applied the principle of randomized controlled trials and no study reported information on allocation concealment and blinding. Therefore, the findings need to be interpreted with caution.

## Investigations on Chinese Herbal Medicine-Derived Compounds Icaritin and Ginsenoside Rg3 Against Primary Liver Cancer

CHM-derived compounds, the active ingredients in CHM, are an important resource for new drug development and have now become a common focus of the research and development of drugs to prevent PLC. Many CHM-derived compounds have been validated to inhibit the development of PLC in *in vivo* and *in vitro* experiments (Bishayee et al., 2010; Hu et al., 2013; Dai et al., 2019; Zhang et al., 2019; Dai et al., 2020). However, translation of academic research into clinical applications is difficult, and many CHM-derived compounds have not yet or have only entered initial clinical trials. At present, ginsenoside Rg3 is undergoing phase III clinical trials on the treatment of PLC, and icaritin (ICT) was approved by the National Medical Products Administration of China on 20 January 2022 for the treatment of HCC (China NMPAo, 2022). ICT and ginsenoside Rg3 have become the CHM-derived compounds with the most potential for clinical application. This section aims to provide an up-to-date report on the mechanisms of action of ICT and ginsenoside Rg3 to facilitate the discovery of new liver cancer drugs from CHMs. We propose that these novel findings may have important implications for PLC clinical treatment and the modernization of CHM for PLC treatment.

### Icaritin

ICT is an active flavonoid extracted from the CHM *Epimedium brevicornum maxim*. E. *brevicornum* maxim is a plant of the *Epimedium* genus in the Berberidaceae family. It was first recorded in Shennong’s Herbal Classic. ICT is prepared by the hydrolysis of icariin, the main active monomer of present of E. *brevicornum maxim* (Ma et al., 2011). Its molecular formula is C_21_H_22_O_7_, and its molecular weight is 368.38. ICT has a wide range of biological activities and has many pharmacological effects, such as antitumor (Yang et al., 2019; Zhang C. et al., 2020), neuroprotective (Li et al., 2020; Tang et al., 2020), cardiovascular protective (Zhang et al., 2016; Ren et al., 2019), immune regulatory (Li et al., 2012; Tao et al., 2021), and bone protective (Wang et al., 2016; Wu et al., 2017), effects. ICT inhibits various cancers to different degrees (Zheng et al., 2014; Wang et al., 2017; Yang et al., 2017) and has been registered as a candidate drug for the treatment of liver cancer by Chinese researchers, becoming a popular research topic in the treatment of cancer.

ICT inhibits cell growth and promotes apoptosis in several HCC cell lines (Table 4). In HepG2 and SMMC7721 cells, ICT downregulates AFP expression and upregulates p53 expression at both the mRNA and protein levels. AFP not only is a target for HCC treatment but also promotes tumor evasion of immune surveillance (Li et al., 2021). ICT also upregulates Bax and downregulates Bcl-2, leading to an elevated Bax/Bcl-2 ratio and triggering the apoptosis of HepG2 cells. Interestingly, ICT activates JNK1 in HepG2 cells, but has no effect on JNK2, ERK or p38, other members of the MAPK family. Thus, ICT exerts its effect by activating JNK1 through a MAPK independent pathway (He et al., 2010). Furthermore, ICT inhibits the activity of sphingosine kinase 1 (SphK1), a critical protein for maintaining the sphingolipid metabolite balance in HCC cells, leading to the production of pro-apoptotic amide and the activation of JNK1 (Lu et al., 2017). Furthermore, ICT is considered a natural glycolysis inhibitor that inhibits GLUT1 expression and the Warburg effect by upregulating the lncRNA target FAM99A and blocking the JAK2/STAT3 pathway (Zheng X. et al., 2021). Another study reported that low concentrations of ICT stimulate reactive oxygen species (ROS) production to mediate the DNA damage response in HepG2 and Huh7 cells, causing them to lose their proliferative potential and exhibit a significant cellular senescence phenotype, as indicated by the significant accumulation of cells in G0/G1 phase with a corresponding decrease in the numbers of cells in S phase and G2/M phase (Wang S. et al., 2019). In addition, ICT downregulates the IL-6/JAK2/STAT3 pathway activity to inhibit hepatocellular carcinoma-initiating cells (HCICs) and effectively reduces the expression of HCIC markers, such as EpCAM, CD133, and CD24, in a dose-dependent manner (Zhao et al., 2015). In addition, ICT exerts an immunomodulatory effect on liver cancer cells by directly binding to and acting on the target proteins MyD88 and IKKα in the TLR/NF-kB signaling pathway to regulate inflammation and the immunomodulatory IL-6/JAK/STAT3 signaling pathway (Zhao et al., 2015). Thus, ICT regulates multiple biological functions of different immune cells and tumor cells and the tumor immune microenvironment, promoting tumor cell apoptosis, inhibiting tumor cell growth, inhibiting the expression of the inflammatory factors IL-6, IL-8, IL-10, and TNF-α, and inhibiting the expression of the immune checkpoint PD-L1 (Mo et al., 2021). Moreover, the synergistic effect of ICT and adriamycin reshapes an immunosuppressive tumor microenvironment and triggers a strong immune memory response, leading to immunogenic cell death (ICD) by inducing mitochondrial autophagy and apoptosis (Yu et al., 2020). In addition, ICT is a novel and effective multidrug resistance reversal agent. In multidrug-resistant HepG2/adriamycin (HepG2/ADR) cell lines, ICT reduces the expression of MDR1 and P-glycoprotein, and reverses resistance to ADR (Sun et al., 2013).

**TABLE 4:** *In vitro* effects of icaritin on liver cancer cells and the underlying mechanisms.

Cell lines	Effective dose/dose range		Effects and related mechanisms	References
HepG2, SMMC7721	20 µM		Inhibits proliferation; Promotes apoptosis; p53↑; AFP↓	Li et al. (2021)
HepG2	5–50 µM		Promotes apoptosis; BAX↑; Bcl-2↓; Bax/Bcl-2↑; caspase-3↑; JNK1↑	He et al. (2010)
Huh-7, KYN-2, Primary human HCC cells	1–25 µM		Increases cellular ceramide production; SphK1↓; JNK1↑	Lu et al. (2017)
HepG2, HCCLM3	2.5, 5, 10 µM		Inhibits proliferation; Inhibits the Warburg effect↓; GLUT1↓; FAM99A↑; JAK2/STAT3↓	Xia Zheng et al. (2021)
HepG2, Huh7	1, 2 µM		ROS↑; Cell cycle arrest in G0/G1 phase↑; Percentage of cells in S and G2/M phases ↓	Shikang Wang et al. (2019)
PLC/PRF/5, Huh7, Hep-12	5–20 µM		EpCAM^+^↓; Stat3↓; Jak2↓; Mcl-1↓; CyclinD1↓; gp130↓; gp80↓; IL-6Rs↓; BMI-1↓; Oct4↓	Zhao et al. (2015)
SMMC-7721	5–20 µM		PD-L1↓; IKK-α↓; Blocking the formation of the IKK‐α/‐β complex; Inhibits p65 translocation; NF‐κB↓	Mo et al. (2021)
Hepa1-6, Huh7		10, 20, 40 µM	Promotes mitophagy; Induces immunogenic cell death; caspase3↑; LC3↑; Atg5↑; Atg7↑; p65↓; PINK1↑; Parkin↑	Yu et al. (2020)
HepG2, HepG2/ADR		1, 15, 30 µM	Reverses multidrug resistance; MDR1↓; P-glycoprotein↓	Sun et al. (2013)

The potential role of ICT has also been documented in orthotopic, subcutaneous and severe combined immunodeficiency (SCID) *in vivo* models of HCC (Table 5). ICT effectively reduces the tumor weight and volume and prolongs survival in an HCC cell-bearing *in vivo* SCID model by inhibiting SphK1 and IL-6/JAK/STAT3 (Zhao et al., 2015; Lu et al., 2017). Another series of *in vivo* studies showed that ICT inhibits HCC development by modulating the tumor immunosuppressive microenvironment, downregulating tumor associated splenic extramedullary hematopoiesis (EMH), significantly reducing tumor and splenic myeloid-derived suppressor cell (MDSC) accumulation and activation, and increasing the number and killing capacity of CD8+T cells (Tao et al., 2021). Furthermore, flow cytometry results showed that the PD -L1^+^ MDSC to CD45^+^ cell ratio and MDSC to CD45+cell ratio were downregulated in BALB/c mice bearing tumors derived from H22 cells (Qin et al., 2020), while ICT did not change the frequency of tumor-infiltrating B cells or NK cells. In addition, when ICT was used as an immunomodulator in combination with an PD-1 antibody, it enhanced the effect of the anti-PD-1 agent in the treatment of liver cancer and reduced the expression of PD-L1 in tumor tissues (Mo et al., 2021; Tao et al., 2021). Excitingly, some researchers found that ICT combined with doxorubicin at a 1:2 ratio had a greater effect than monotherapy, and the combination therapy reduced the immunosuppressive function of MDSCs, regulatory T (Treg) cells, and M2 macrophages; decreased the release of immunosuppressive cytokines, such as CCL2, TGF β, IL-4, IL-6, and IL-10; increased the numbers of CD8^+^ T cells, CD4+T cells, and activated dendritic cells (DC cells); increased IFN-γ, TNF-α, and IL-12 antitumor activities and functions and ultimately induced ICD (Yu et al., 2020).

**TABLE 5:** Biological effects of icaritin on *in vivo* models of HCC.

Models	Administration	Effective doses	Effects/related mechanisms	References
SCID mice implanted with HepG2 cells	Administered by gastric gavage for 30 days	2.5, 10 mg/kg	Prolongs Mouse survival; Inhibits HepG2 tumor growth; SphK1↓	Lu et al. (2017)
NOD/SCID mice implanted with PLC/PRF/5 and Hep-12 cells	Administered by gastric gavage for 14 days	17.5, 70 mg/kg	Inhibits tumor growth; Reduces tumor occurrence; Stat3↓; Mcl-1↓; Jak2↓	Zhao et al. (2015)
Hepa1-6 cells injected into the left lobe of the liver of C57BL/6 mice; Hepa1-6 or H22 cells injected into the right flank of C57BL/6 mice or BALB/c mice	Administered by gastric gavage for 3 weeks	70 mg/kg	Suppresses tumor growth; Prolongs survival; Increases the total number and activity of CTLs; Reduces the total number and activity of tumor-infiltrating PMN-MDSCs; Reduces the PMN-MDSC frequency and increases the CTL frequency in the spleen; Decreases the accumulation of myeloid-biased HSPCs in the spleen; Upregulates PD-L1 expression on tumoral and splenic PMN-MDSCs; IFN-γ+CD3+CD8+CTL↑; Arg-1↓; Stat3↓	Tao et al. (2021)
C57BL/6 mice bearing Hepa1‐6 tumors	Administered by gastric gavage for 20 days	70 mg/kg	Decreases tumor volume and tumor weight; PD-L1↓	Mo et al. (2021)
BALB/c mice bearing H22 cells tumors	Administered by gastric gavage	70 mg/kg	Inhibits tumor growth; PD‐L1^+^ MDSCs to CD45^+^ ratio↓; MDSCs to CD45^+^ ratio↓	Qin et al. (2020)
Immune-competent C57BL/6 mice injected with Hepa1–6 cells in a hemisplenic model	PLGA NP encapsulated Icaritin and combination every other day for a total of 4 injections	Icaritin (5 mg/kg), combination (icaritin: 1 mg/kg, doxorubicin: 3 mg/kg)	Remodels the immune-suppressive microenvironment; CD8^+^ T cells↑; CD4^+^ T cells↑; Activated DC cells↑; Memory T cells↑; MDSCs↓; Tregs↓; M2 macrophages↓; IFN-γ↑; TNF-α↑; IL-12↑; CCL2↓; TGF-β↓; IL-4↓; IL-6↓; IL-10↓	Yu et al. (2020)

A phase I/II clinical trial of ICT has been completed, and two phase III studies are currently underway in China (Table 6). The phase I clinical study enrolled 20 patients with advanced HCC, and the results showed that ICT therapy was well tolerated and had good safety, with no drug-related grade 3/4 adverse events (AEs) or immune-related AEs observed, and the treatment modulated serological immune inflammatory indexes in patients with advanced HCC (Fan et al., 2019). Sixty-eight patients with advanced HCC (91.2% with HBV infection) were recruited from five hospitals in China in a phase II clinical trial, and patients with HCC presenting a stable disease (SD) who experienced long-term ICT administration exhibited a good and durable survival rate, with a mOS of 496 and 383.5 days after treatment for more than 135 days, and less than 135, respectively; in addition, the treatment showed a significant safety advantage over days, traditional targeted agents (Qin et al., 2020). Two phase III trials are currently recruiting, and NCT03236649 is enrolling patients with PD-L1-positive advanced HCC to compare the safety and efficacy of ICT versus sorafenib (Sun and Qin, 2021a). NCT03236636 was mainly focused on Chinese patients with HCC in terms of protocol design, 71 of 283 included patients with advanced HCC had positive CBS scores (AFP≥400 ng/ml, TNF-a<2.5 pg/ml and IFN-g ≥7.0 pg/ml), along with unfavorable prognostic factors such as BCLC C stage disease, HBV infection, and thrombocytopenia, which are more common in Chinese patient populations with HCC. The interim results were presented at the 2021 American Society of Clinical Oncology annual meeting by the investigators of the trial: a significantly higher mOS of 13.54 months was achieved with ICT in the composite biomarker score (CBS) enriched population, which was significantly longer than the 7.06 months achieved with Huachansu Pian (Sun and Qin, 2021b; Sun et al., 2021). National Medical Products Administration of China approved ICT soft capsule for marketing on 20 January 2022. ICT soft capsules are indicated for patients with unresectable HCC who are not eligible for or refuse standard treatment and have not received prior systemic therapy. In Addition, the serological tests of HCC patients met at least two of the following indicators: AFP ≥400 ng/ml, TNF- α < 2.5 pg/ml, IFN- γ ≥ 7.0 pg/ml (China NMPAo, 2022). In conclusion, ICT has shown a better safety profile in clinical experiments and prolongs the survival of patients with advanced HCC who have a poor prognosis, but high-quality clinical experimental validation is still needed.

**TABLE 6:** Effects of icaritin on HCC evaluated in clinical studies.

Clinicaltrials. gov Identifier	Phases	Condition	Participants (M/F)	Interventions	Results	References
NCT02496949	Phase I	Advanced HCC	20 (17/3)	Icaritin: 600 mg/dose or 800 mg/dose, 2 doses/day, oral administration 28-days treatment cycle	Safety, regulation of immunokinetics and biomarkers	Fan et al. (2019)
NCT01972672	Phase II	Advanced HCC	68 (61/7)	Icaritin: 600 mg/dose or 800 mg/dose, 2 doses/day, oral administration; Continuous until disease progression, intolerable toxicity, and/or patient discontinuation	Favorable and durable survival, Immunomodulation	Qin et al. (2020)
NCT03236636	Phase III	HCC	Estimated 312	Icaritin: 600 mg/dose, 2 doses/day, oral administration Huachansu Pian: 1.2 g/dose, 3 doses/day, take oral administration; Continuous administration until the standard of termination is reached	Recruiting	Sun and Qin, (2021b)
NCT03236649	Phase III	HCC	Estimated 200	Icaritin: 600 mg/dose, 2 doses/day, oral administration Sorafenib tosylate tablets: 400 mg/dose, 2 doses/day, oral administration; Continuous administration until the standard of termination is reached	Recruiting	Sun and Qin, (2021a)

### Ginsenoside Rg3

Ginsenoside Rg3 is a tetracyclic triterpenoid saponin, that is one of the main components isolated from the TCM ginseng. Based on the spatial position of the C-20 hydroxyl group, it is divided into 20 (R) and 20 (S) isomers (Kim I. W. et al., 2013). The two isomers of ginsenoside Rg3 differ in pharmacokinetics and bioactivity; 20 (R)-ginsenoside Rg3 has a much shorter half-life than 20 (S)-ginsenoside Rg3, Intravenous administration in rats or oral administration of 20 (R) ginsenoside Rg3 (50 mg/kg) produced a plasma, concentration that was almost undetectable throughout the sampling period, but 20 (S) ginsenoside Rg3 was detected because 20 (R)-ginsenoside Rg3 underwent single direction chiral inversion into 20 (S)-ginsenoside Rg3. This finding suggests that ginsenosides may exert their effects on rat plasma via 20 (S)-ginsenoside Rg3 (Peng et al., 2016). The general tolerability of 20 (S)-ginsenoside Rg3 in humans has been good, with a time to maximum plasma concentration achieved by intramuscular injection every 4 h and a urinary excretion rate of less than 1% for the dose over 72 h, achieving a pharmacokinetic profile suitable for administration of once every 2 days (Zhao et al., 2016). Compared with 20 (R)-ginsenoside Rg3, 20 (S)-ginsenoside Rg3 more strongly inhibited hepatoma cell viability and induced a more significant reduction in global genomic DNA methylation (Teng et al., 2017). Thus, 20 (S)- ginsenoside Rg3 has been revealed as the better choice for drug development.

Ginsenoside Rg3 significantly inhibits the growth of a variety of liver cancer cell lines, including HepG2, SK-Hep1, MHCC-97L, MHCC-97H, SMMC-7721, and BEL-7404, and effectively inhibits the migration and invasion of liver cancer cells by upregulating ARHGAP9 protein expression (Sun et al., 2019). Furthermore, ginsenoside Rg3 has been reported to induce G1 arrest during the cell cycle progression of HCC cells, which is associated with decreased expression of CDK2, cyclin D1, PCNA, and SIRT2 and increased expression of H3K18ac and H4K16ac (Li et al., 2018; Shan et al., 2019; Zheng Q. et al., 2021). In addition, ginsenoside Rg3 not only reduces the expression and activity of NHE1 by inhibiting EGF-EGFR-ERK1/2-HIF-1 alpha signaling (Li et al., 2018) but also activates the mitochondria-mediated caspase cascade, leading to the loss of the mitochondrial membrane potential. Ginsenoside Rg3 stimulates the release of cytochrome c from the mitochondrial membrane space into the cytosol, activates the caspase-3 and Bax proteins, and inhibits the production of Bcl-2 protein and intracellular ROS (Jiang et al., 2011; Park et al., 2012; Zhang et al., 2012). Interestingly, the cleavage of caspase-8 was not obvious, suggesting that ginsenoside Rg3 does not activate caspase-8 to induce apoptosis through the death receptor pathway in liver cancer cells (Park et al., 2012). Ginsenoside Rg3 also inhibit angiogenesis by reducing the expression of VEGF (Yu et al., 2013; Teng et al., 2017). When ginsenoside Rg3 is used in combination with chemotherapy drugs, it upregulates CHOP-mediated DR5 expression at the transcriptional level, exerting a synergistic effect (Lee et al., 2013) (Table 7).

**TABLE 7:** *In vitro* effects of ginsenoside Rg3 on liver cancer cells and the underlying mechanisms.

Cell lines	Effective dose/dose range	Effects and related mechanisms	References
SMMC-7721, HepG2	50, 100 μg/ml	Inhibits proliferation; Promotes apoptosis; caspase-3↑; Bax ↑; Bcl-2↑	Zhang et al. (2012)
Bel-7402, HCCLM3	10, 25, 50, and 100 μM	Reduces cell viability; Induces cell apoptosis, G1↑; S phase ↓; Cleaved-caspase-3↑; NHE1↓; EGFR↓; EGF↓; HIF-1α↓; ERK1/2↓	Li et al. (2018)
SMMC-7721	7.5, 15, 30, 60, and 100 μg/ml	Inhibiting proliferation; Promote apoptosis; Cyclin D1↓; PCNA↓	Shan et al. (2019)
Bel-7402, HCCLM3	40, 80, 160 µM	Reduces cell viability; Number of cells in G1 phase ↑; CDK2 ↓; Cyclin D1↓; SIRT2↓; H3K18 ac↑; H4K16ac↑	Qiyu Zheng et al. (2021)
HepG2, SK-Hep1, MHCC-97L, MHCC-97H, SMMC-7721, BEL-7404	1.25, 2.5, 5 μg/ml	Reduces cell viability; Blocks invasion and migration; ARHGAP9↑	Sun et al. (2019)
Hep1-6, HepG2	50, 100, 200 μg/ml	Reduces cell viability; Induces apoptosis; caspase-3 ↑; Cyto (c) ↑; Cyto (m) ↓; Bcl-2↓; Bcl-XL↓; Bax↑	Jiang et al. (2011)
Hep3B	1, 3, 10, 30, 50 µM	Promotes apoptosis; ROS↑; caspase-3↑; Cyto (c) ↑; Bax↑; Bcl-2↓	Park et al. (2012)
HepG2	100, 200, 300, 400, 500 μg/ml	Global genomic DNA methylation↓; DNMT1↑; DNMT3a↓; DNMT↓; P53 ↑; Bcl-2 ↓; VEGF↓	Teng et al. (2017)
HepG2	25, 50, 75, 100 mg/L	Inhibits cell viability; VEGF↓	Yu et al. (2013)
SK-Hep1, HepG2, Hep3B, HL-7702	100 μM	caspase-3↑; PARP↑; DR5↑; CHOP↑; EIF2α↑; GRP78↑	Lee et al. (2013)

*In vivo* studies have revealed that direct targeted inhibition of VEGF by ginsenoside Rg3 in combination with TACE blocked angiogenesis and tumor growth, eventually reducing the tumor size and metastasis and prolonging OS in a buffalo rat orthotopic liver cancer model and rabbit liver VX2 carcinoma models (Yu et al., 2013; Zhou et al., 2014). The inhibitory effect of ginsenoside Rg3 on tumor MVD was also observed in C57BL/6 mice implanted with Hepa1-6 cells, and ginsenoside Rg3 initiated apoptosis of the tumor cells (Li et al., 2018), decreased the tumor volume and ability of the tumor to generate vascularized networks, and inhibited further growth and remote metastasis (Hu et al., 2019). Ginsenoside Rg3 has shown good antitumor activity *in vivo* and is well tolerated without significant toxic side effects, but it has low water solubility and poor oral bioavailability, which affect clinical applications. Investigators have conjugated Fe@Fe_3_O_4_ nanoparticles with ginsenoside Rg3 (NpRg3) to develop a nanomedicine that achieves an excellent conjugation effect and improved antitumor function. In a dimethylnitrosamine-induced HCC model, NpRg3 prolonged the survival time of HCC mice and abolished the metastasis of HCC to the lung. Notably, NpRg3 inhibits liver cancer development and metastasis by remodeling the imbalanced intestinal flora and metabolism, increasing the abundance of Bacteroidetes and Verrucomicrobia, but decreasing the abundance of Firmicutes. NpRg3 corrected the predominant tumor metabolomic profile by decreasing 3-indolepropionic acid and urea levels, and increased free fatty acids levels, suggesting that this agent might be a new treatment for liver cancer (Ren et al., 2020) (Table 8).

**TABLE 8:** Biological effects of ginsenoside Rg3 on *in vivo* models of HCC.

Models	Administration	Effective doses	Effects/related mechanisms	References
DEN-induced HCC in C57BL/6 mice	Oral administration 5 times from 29 to 30 weeks	70 mg/kg	Prolongs the survival time; Reduces the number of liver surface tumors; Decreases lung metastasis; Remodels the unbalanced networks between gut microbiota and metabolism; Inhibits the morphological changes in the ileocecal part; Enhances the immune response; Bacteroidetes↑; Verrucomicrobia↑; Firmicutes↓; 3-indolepropionic acid↓; urea↓; free fatty acids↑; ALT ↓; AST↓; FFA 18_2↑; FFA 16_2 ↑; N8-acetylspermidine↓; creatinine↓	Ren et al. (2020)
C57BL/6 mouse implanted with Hepa1‐6 cells	Oral administered for 30 days	10 mg/kg	Prolongs the survival time; Maintains weight; Reduces the tumor weight; Induces apoptosis; Inhibits angiogenesis	Hu et al. (2019)
BALB/c nude injected with HCCLM3 cells	Intraperitoneally injected for once every 2 days for 3 weeks	10 mg/kg	Suppresses tumor growth; Ki67↓; Cleaved-caspase-3↑; NHE1↓; EGFR↓; EGF↓; HIF-1α↓; ERK1/2↓	Li et al. (2018)
Buffalo rat implanted with McA-RH7777 cells	Hepatic arterial infusions of normal saline or iodized oil (0.1 ml/kg) with ginsenoside Rg3	1 mg/kg	Reduces the longest tumor diameter; Maintains body weight; Blocks tumor metastasis; Prolongs survival; MVD↓; CD31↓; VEGF↓; VEGF-R2↓	Zhou et al. (2014)
Rabbit liver VX2 carcinoma models	Ginsenoside Rg3, lipiodol (0.1 ml/kg) or a mixture of both was injected through the catheter until a reduction in blood flow to the tumor was observed	6.0 mg/kg	Inhibits tumor growth; CD31↓; VEGF↓; caspase-3 ↑; Bax↑	Yu et al. (2013)

NCT01717066, a phase II, multicenter, trial, randomized, double-blind, placebo-controlled study in 480 patients with stage 1 and 2 HCC, was performed to evaluate the efficacy and safety of ginsenoside Rg3 in preventing postoperative recurrence with undisclosed results (Shen, 2021). However, a phase III, single center, open label, randomized controlled trial (ChiCTR-TRC-11001643) with published results was registered in the Chinese clinical trial registry. The trial compared the efficacy and safety of ginsenoside Rg3 in combination with TACE *versus* TACE alone in patients with advanced HCC. Among 259 patients with advanced HCC and Child-Pugh A liver function, ginsenoside Rg3 combined with TACE prolonged the median survival by 3.1 months compared to TACE alone (13.2 vs. 10.1 months, P = . 002). It also significantly improved the DCR (69.7% vs. 51.3%, *p* = 0 0.012) and 12-months OS rate (54.6% vs. 35.5%, *p* = 0.008). Although no significant difference in the 6-months OS rate, the median time to progression or median time to nontreatable progression (TTUP) were observed, some TACE-associated adverse syndromes and hematological abnormalities were alleviated by the combination therapy. Therefore, ginsenoside Rg3 is an adjuvant treatment with certain efficacy and superior tolerability. However, the study only enrolled patients with HCC presenting Child-Pugh A liver function, and patients with Child-Pugh B or C liver function were not evaluated. Thus, the value of ginsenoside Rg3 in the comprehensive treatment of HCC requires further investigation (Zhou et al., 2016).

## Perspectives and Challenges

The progress in drug development for PLC is relatively slow and the current demand for first-line treatment is far from being met; therefore, an exploration of alternative strategies to treat PLC is urgently needed. CHM represents a new alternative strategy for the treatment of PLC, expanding the therapeutic options for PLC. This review introduced clinical studies of CPMs and CHM-derived compounds for the treatment of PLC and the underlying mechanisms, providing inspiration for researchers investigating natural medicines. However, to date, CPMs for the treatment of PLC have been assessed in clinical studies with poor quality design and methods and high risks of bias, and most reports of CHM-derived compounds have also been solely performed in cell and animal models. In addition, CHMs face many challenges, such as low bioavailability and hepatotoxicity. The dilemmas of potential toxicity and low bioavailability must be solved, and evidence-based medicine from large-sample, long-term clinical trials is also needed to ensure the efficacy and safety of CHMs and better translate the findings into clinical practice.

The safety of CHMs is a key factor for their clinical application (Opara, 2004). However, CHM ingredients are complex that exert effects by targeting multiple factors and pathways, and the interaction between ingredients is not clear, resulting in difficulty in determining the toxicity of CHM substances. Researchers should conduct structural experiments as early methods to predict the toxic components of CHMs, use comprehensive analytical pharmacokinetics and toxicology, and strengthen *in vivo* and *in vitro* experiments to more comprehensively reveal the toxicity and mechanisms of CHMs (Wang et al., 2009). In addition, a system to evaluate the safety of CHMs specifically must be established, not only to exclude exogenous pollution factors such as pesticide residues, heavy metals, and fungi, but also to assess the specific foreign bodies, origin and cultivation conditions, mechanism of action, compatibility, and other factors of each CHM (Luo et al., 2019). Furthermore, investigators should extend the clinical monitoring of adverse reactions and toxic side effects of CHMs, focus on modern toxicology research reports and strengthen data analysis to reduce and prevent the occurrence of AEs of CHMs and improve the safety of clinical medications (Ma et al., 2014).

The most common route of administration for CHMs is oral administration. However, many active ingredients seriously affect the clinical efficacy of CHMs because of their poor oral absorption and low bioavailability. The reasons for the poor bioavailability of orally administered drugs mainly include poor drug solubility and a low dissolution rate. The gastrointestinal mucosa is poorly permeable to some drugs and the drugs do not readily permeate through biological membranes, leading to lower drug absorption (Kesarwani et al., 2013). Therefore, structural modification of CHMs to improve their physicochemical characteristics or the use of new preparation methods to prepare formulations with high bioavailability may improve the application value of CHM against liver cancer to some extent. Amorphous ICT nanoparticles prepared using a reactive precipitation technique and ICT-loaded polymeric micelles prepared by using the acid-base Schift method were able to effectively deliver ICT across the intestinal epithelium and improve the oral bioavailability of poorly water-soluble ICT (Tang et al., 2021a; Tang et al., 2021b). Novel ginsenoside liposomes prepared by the thin film hydration method not only act as membrane stabilizers with long blood circulation times but also serve as active targeting ligands to home onto cancer cells and greatly improve the antitumor efficacy and reduce the side effects of ginsenosides due to off-target effects (Hong et al., 2019). Furthermore, CHM formulae contain many herbs, and a single preparation method may not meet the conditions ideal for the individual herbs in CHM formulations. Producing multiple active components with different properties in the CHM formulations into different prodrugs, can change the release behavior of these active components and affect their *in vivo* processes. This strategy, might exert a greater clinical therapeutic effect by activating of different pathways and targets, reducing toxic side effects, and improving the bioavailability of CHM formulas, which will undoubtedly be a focus of modern CHM formulation research.

## Conclusion

In summary, CPMs Huaier granules, GFL, FFBM, JLC, BJOE, and CKI can prolong the survival and improve the quality of life of patients with PLC through multiple pathways and targets. CHM-derived compounds ICT and ginsenoside Rg3 have been validated to be effective against PLC in *in vivo*, *in vitro*, clinical studies and showed great potential for clinical application. However, the hepatotoxicity and low bioavailability of oral CHMs remain as challenges that still need to be overcome. Therefore, more experiments and studies exploring these topics are still needed.
